# Diagnostic performance of conventional optical coherence tomography parameters for differentiating pediatric papilledema from pseudopapilledema

**DOI:** 10.1186/s12886-026-05420-5

**Published:** 2026-10-17

**Authors:** Selda Celik Dulger, Aykut Karatas

**Affiliations:** Department of Ophthalmology, Ankara Etlik City Hospital, Halil Sezai Erkut Caddesi No:5, Yenimahalle, Ankara, Türkiye

**Keywords:** Papilledema, Pseudopapilledema, Optic disc drusen, Crowded optic discs, Optical coherence tomography

## Abstract

**Purpose:**

To evaluate the diagnostic performance of conventional optical coherence tomography (OCT)-derived peripapillary retinal nerve fiber layer (RNFL) and macular ganglion cell-inner plexiform layer (GC-IPL) thickness measurements for differentiating papilledema from pseudopapilledema in children.

**Methods:**

This retrospective study included 115 pediatric patients (230 eyes) with papilledema, optic disc drusen (ODD)-related pseudopapilledema, pseudopapilledema without optic disc drusen, and healthy controls. Peripapillary RNFL and macular GC-IPL thicknesses were measured using spectral-domain OCT. Group comparisons were performed using generalized estimating equations, and diagnostic performance was assessed using receiver operating characteristic analysis.

**Results:**

The study included 26 patients (52 eyes) with papilledema, 30 patients (60 eyes) with optic disc drusen (ODD)-related pseudopapilledema, 32 patients (64 eyes) with pseudopapilledema without ODD, and 27 healthy controls (HCs) (54 eyes). Mean pRNFL thickness was 129.85 ± 51.08 μm in the papilledema group, 128.22 ± 36.78 μm in the ODD-related pseudopapilledema group, 106.64 ± 17.10 μm in the pseudopapilledema without ODD group, and 99.89 ± 11.29 μm in the healthy control group. Compared with HCs, RNFL thickness was significantly increased in all quadrants in eyes with ODD-related pseudopapilledema, in three quadrants in papilledema, and only in the superior quadrant in pseudopapilledema without ODD. Compared with HCs, inferior RNFL demonstrated the highest discriminatory ability for papilledema (AUC = 0.848, *p* < 0.001) and ODD-related pseudopapilledema (AUC = 0.906, *p* < 0.001), whereas superior RNFL showed significant discrimination for pseudopapilledema without ODD (AUC = 0.720, *p* = 0.001). For differentiating papilledema from ODD-related pseudopapilledema, nasal RNFL yielded the highest AUC among the evaluated RNFL parameters (AUC = 0.699, *p* = 0.005). For papilledema versus pseudopapilledema without ODD, inferior RNFL demonstrated the highest diagnostic performance (AUC = 0.732, *p* < 0.001), followed by temporal RNFL (AUC = 0.710, *p* = 0.002). Several GC-IPL parameters demonstrated significant discriminatory ability in specific comparisons, with inferonasal GC-IPL showing the highest AUC for ODD-related pseudopapilledema versus healthy controls (AUC = 0.731, *p* = 0.004) and superotemporal GC-IPL showing the highest AUC for papilledema versus ODD-related pseudopapilledema (AUC = 0.769, *p* = 0.001).

**Conclusions:**

Conventional OCT-derived RNFL measurements demonstrated limited diagnostic performance for distinguishing pediatric papilledema from pseudopapilledema, particularly in eyes with ODD-related pseudopapilledema. OCT findings should therefore be interpreted together with clinical assessment and, where available, advanced optic nerve head imaging techniques.

## Introduction

Suspected papilledema is one of the most common reasons for referral to neuro-ophthalmology clinics in children. However, only a small proportion of pediatric patients referred with suspected papilledema are ultimately diagnosed with true papilledema [1]. Misclassification may lead to unnecessary investigations, including neuroimaging and lumbar puncture. Therefore, establishing a reliable and noninvasive method for distinguishing true papilledema from pseudopapilledema is critical in pediatric practice.

In children, pseudopapilledema most commonly results from optic disc drusen (ODD), and peripapillary hyperreflective ovoid mass-like structures (PHOMS) are frequently associated with ODD but may also occur in other optic nerve head disorders. Enhanced depth imaging optical coherence tomography (EDI-OCT) has become an important modality for visualizing optic nerve head architecture and identifying structural features such as PHOMS. These structures are frequently observed in children with optic disc drusen but may also occur in papilledema and other optic nerve head anomalies. Therefore, PHOMS should not be considered a specific marker for differentiating papilledema from pseudopapilledema. Nevertheless, B-scan ultrasonography, fundus autofluorescence, and conventional spectral-domain OCT continue to play an important complementary role in routine clinical practice, particularly in pediatric patients in whom image acquisition with EDI-OCT may be challenging [2–5]. Recent studies have increasingly emphasized a multimodal approach for differentiating papilledema from pseudopapilledema, integrating clinical evaluation with ancillary imaging modalities such as EDI-OCT, fundus autofluorescence, B-scan ultrasonography, and optic nerve head assessment. Nevertheless, no single imaging modality is sufficient in all cases, particularly in children, and each technique has inherent limitations [2, 5–8]. 

Optical coherence tomography (OCT) is a noninvasive and widely available imaging modality that provides quantitative assessment of the optic nerve head and retina. Conventional spectral-domain OCT, particularly peripapillary retinal nerve fiber layer (RNFL) thickness, remains one of the most widely used quantitative OCT parameters in routine pediatric neuro-ophthalmic practice. RNFL thickness is widely used for evaluating optic disc swelling; however, increased RNFL thickness has been reported in both papilledema and pseudopapilledema, resulting in substantial overlap between these conditions and limiting its diagnostic accuracy, particularly in children and in eyes with buried optic disc drusen. The precise diagnostic performance of conventional RNFL measurements remains uncertain [5, 8–12]. 

Macular ganglion cell-inner plexiform layer (GC-IPL) thickness has been widely used for evaluating optic neuropathies. However, its role in differentiating pediatric papilledema from pseudopapilledema remains unclear, with previous studies reporting inconsistent findings [13–16]. 

The aim of this study was to evaluate the diagnostic performance of conventional OCT-derived peripapillary RNFL and macular GC-IPL measurements for differentiating papilledema from ODD-related pseudopapilledema and pseudopapilledema without ODD in a pediatric population.

## Materials and methods

This nonrandomized, retrospective, single-center clinical study was approved by the Ethics Committee of a tertiary hospital (Ethics number: AEŞH-BADEK-2025-135, 14.05.2025) and conducted in accordance with the tenets of the Declaration of Helsinki. Written informed consent was obtained from all participants aged 16 years or older. For participants younger than 16 years, written informed consent was obtained from their parents or legal guardians.

The study population comprised four groups: pediatric patients with papilledema, ODD-related pseudopapilledema, pseudopapilledema without ODD, and healthy controls. Data from the three patient groups who were followed at a tertiary neuro-ophthalmology clinic between January 2023 and December 2024 were retrospectively reviewed, while healthy controls were recruited from the outpatient clinic during the same period. Collected data included demographic characteristics and ophthalmologic findings, including best-corrected visual acuity (BCVA), optic disc appearance, OCT parameters, ocular B-scan ultrasonography (BSUS), and automated perimetry using the 30 − 2 Swedish Interactive Threshold Algorithm (SITA) Standard protocol on the Humphrey Field Analyzer 3 (Carl Zeiss Meditec, Dublin, CA, USA). Optic disc appearance and Frisén grade were obtained retrospectively from the clinical examination findings documented in the hospital information system during the patients’ routine neuro-ophthalmology follow-up examinations. These examinations were performed by the same neuro-ophthalmologist who followed and clinically evaluated the patients. Brain magnetic resonance imaging (MRI), magnetic resonance venography (MRV), and lumbar puncture (LP) findings were recorded when these investigations had been performed as part of the routine clinical evaluation.

**Fig. 1:**
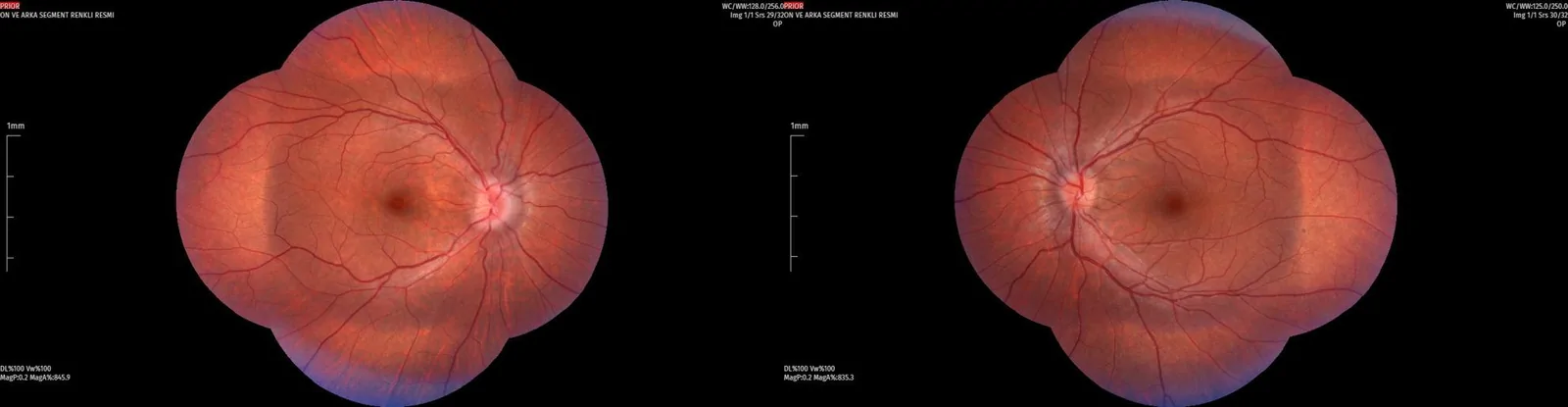
Color fundus photograph of a female patient diagnosed with idiopathic intracranial hypertension **s**howing bilateral Frisén grade 1 papilledema

In the papilledema group (*n* = 26), 22 patients had idiopathic intracranial hypertension (all underwent LP with an opening pressure > 250 mm H₂O) (Fig. 1), three had intracranial hypertension secondary to cerebral venous sinus thrombosis, and one had hydrocephalus, for which a lumboperitoneal shunt was placed.

**Fig. 2:**
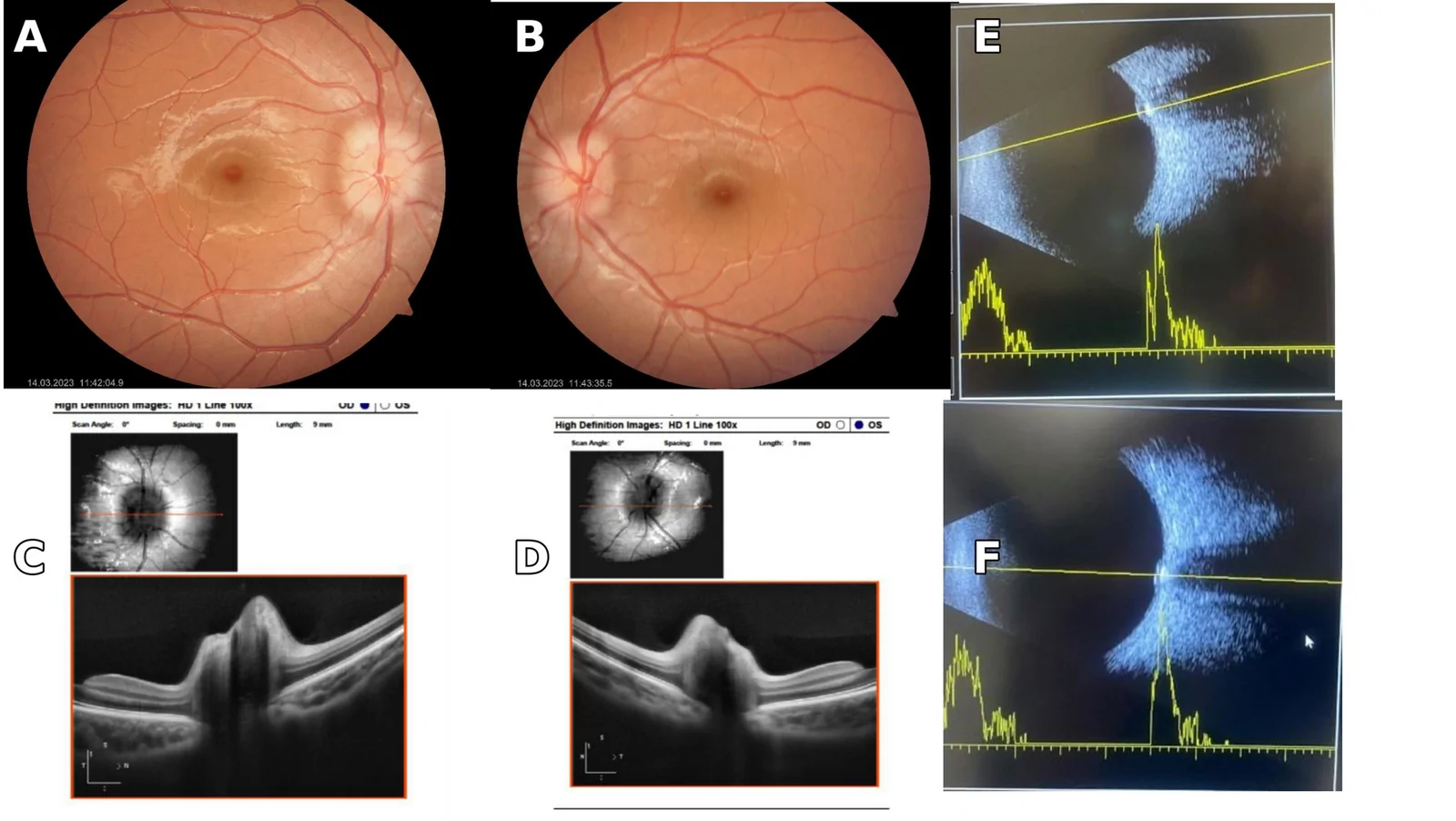
Multimodal imaging findings in a child with optic disc drusen.** (A**,** B)** Color fundus photographs of the right and left eyes, respectively, showing optic disc elevation and blurred disc margins. **(C**,** D)** Optic nerve head OCT images of the right and left eyes, respectively, showing optic disc elevation. **(E**,** F)** B-scan ultrasonography of the right and left eyes, respectively, demonstrating a highly reflective lesion at the optic nerve head consistent with optic disc drusen

The pseudopapilledema group was subdivided into ODD-related pseudopapilledema and pseudopapilledema without ODD. The diagnosis of ODD was established using multimodal clinical and imaging assessment. Characteristic hyperreflective echoes on B-scan ultrasonography (BSUS) were required for the diagnosis in all ODD eyes. Fundus autofluorescence and enhanced depth imaging optical coherence tomography (EDI-OCT), when available, were reviewed for supportive imaging findings, consistent with the imaging principles proposed by the Optic Disc Drusen Studies (ODDS) Consortium [5]. Characteristic hyperreflective echoes on BSUS were present in all eyes classified as ODD (Fig. 2).

The pseudopapilledema group without ODD included patients with congenitally crowded optic discs, no evidence of ODD on B-scan ultrasonography (BSUS), and no supportive fundus autofluorescence or enhanced depth imaging optical coherence tomography (EDI-OCT) findings when these imaging modalities were available, and a stable optic nerve appearance during at least 6 months of follow-up. Neuroimaging (brain MRI with or without MRV) and lumbar puncture were performed when clinically indicated. In patients who did not undergo neuroimaging or lumbar puncture at our institution, the diagnosis was established based on the absence of neurological symptoms or signs, a stable clinical course during follow-up, and review of previous neuroimaging findings when available. None of these patients developed clinical findings consistent with papilledema during follow-up.

Healthy control subjects (*n* = 27) were recruited from patients presenting to the outpatient clinic for conditions not affecting the retina or optic nerve. Inclusion criteria for HCs were BCVA ≥ 20/20, normal slit-lamp and fundus examinations, normal visual fields, a normal-appearing optic nerve head, and no history of ocular, neurologic, or systemic disease.

Exclusion criteria for all participants included incomplete clinical data, comorbid ocular disease or prior ocular surgery, retinal vascular disease, systemic disease, refractive error exceeding ± 6 diopters. Participants who were unable to cooperate adequately to obtain reliable OCT images were excluded. Patients with severe papilledema (Frisén grade 4–5) or signs of chronic papilledema (disc pallor and/or macular exudates) were excluded.

A total of 115 pediatric participants were enrolled, including 26 patients (52 eyes) with papilledema, 30 patients (60 eyes) with ODD-related pseudopapilledema, 32 patients (64 eyes) with pseudopapilledema without ODD, and 27 healthy controls (54 eyes), yielding a total of 230 eyes for analysis.

**Fig. 3:**
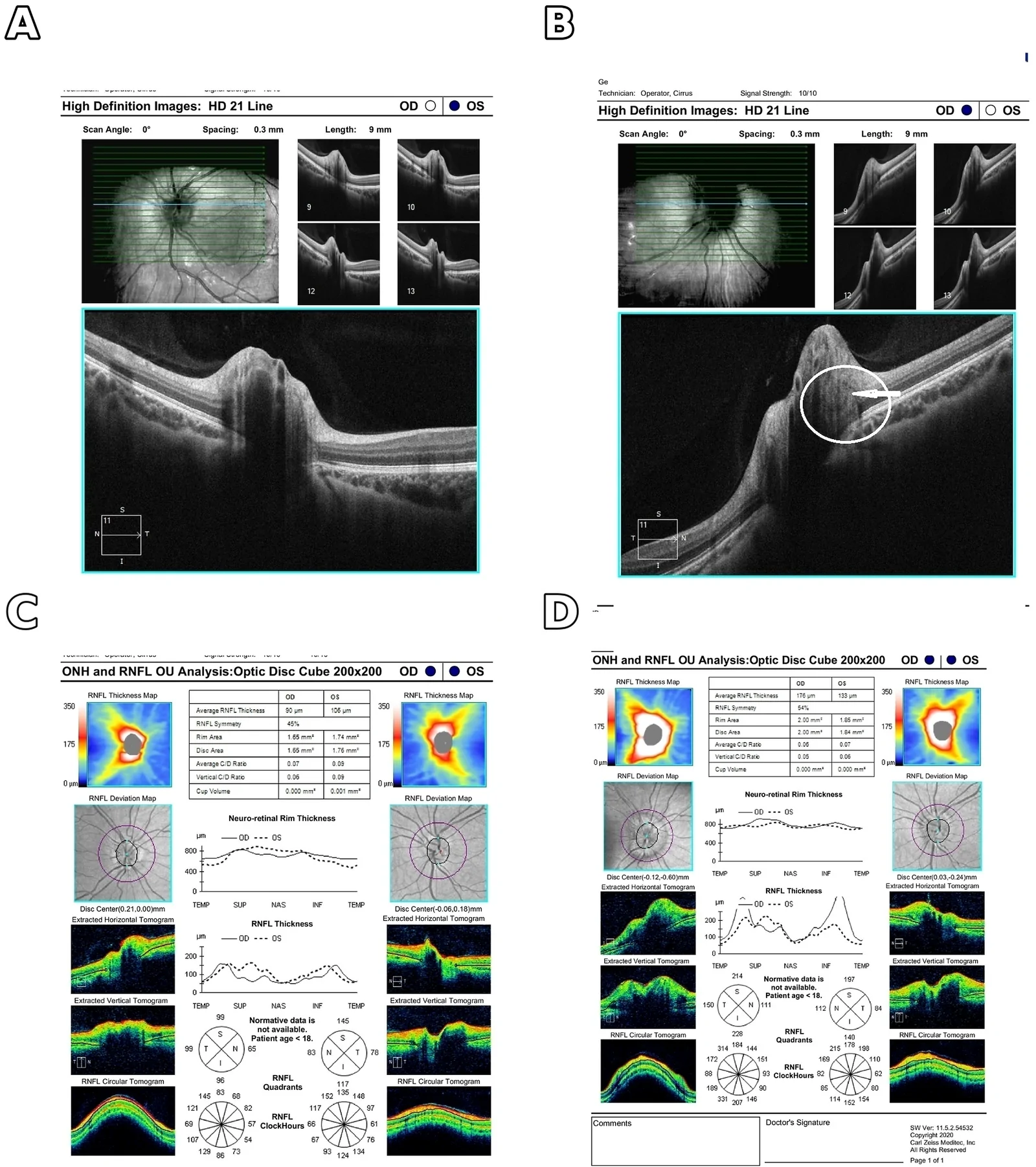
(**A**) Optic nerve head OCT image of the left eye (OS) obtained at baseline. (**B**) Optic nerve head OCT image of the right eye (OD) obtained at baseline, showing a peripapillary hyperreflective ovoid mass-like structure (PHOMS; white arrow and circle). (**C**) RNFL thickness analysis of both eyes obtained at baseline. (**D**) RNFL thickness analysis of both eyes obtained after 6 months of topiramate treatment. The serial RNFL thickness maps demonstrate a reduction in peripapillary RNFL thickness following treatment and are presented as an illustrative longitudinal observation; they were not included in the main quantitative analyses

Peripapillary RNFL and macular GC-IPL images were acquired using Cirrus HD-OCT (Carl Zeiss Meditec, Dublin, CA, USA). RNFL thickness was measured using the Optic Disc Cube 200 × 200 protocol, with a 3.4-mm diameter circular scan centered on the optic disc (Figs. 2 and 3). Macular GC-IPL thickness was measured using the Macular Cube 512 × 128 protocol, providing a quantitative assessment of the combined ganglion cell layer and inner plexiform layer. Optic disc area was obtained directly from the automated output of the Cirrus HD-OCT. Automated segmentation was reviewed for all scans, and scans with segmentation errors were excluded from the analysis; no manual segmentation correction was performed. OCT measurements included in the main analyses were obtained before initiation of treatment in patients with papilledema.

### Statistical analysis

Statistical analyses were performed using IBM SPSS Statistics version 23.0 (IBM Corp., Armonk, NY, USA) and Jamovi version 2.3.28. Normality was assessed using the Shapiro–Wilk and Kolmogorov–Smirnov tests. The Kruskal–Wallis H test was used to compare three or more groups with non-normally distributed data. Both eyes were included in the analyses when eligible. Because measurements from the two eyes of the same patient are not statistically independent, generalized estimating equation (GEE) models were used to compare RNFL and GC-IPL thicknesses between groups while accounting for within-patient inter-eye correlation, with Bonferroni correction applied for multiple comparisons. Descriptive mean ± SD values were calculated at the eye level using measurements from all included eyes. Pearson or Spearman correlation analyses were used to assess associations between visual field mean deviation and mean RNFL and GC-IPL thicknesses, as appropriate. Fisher’s exact test with Monte Carlo correction was applied for categorical variables. Receiver operating characteristic (ROC) curve analysis was used to evaluate the ability of OCT parameters to discriminate papilledema and pseudopapilledema from healthy controls, as well as to differentiate papilledema from pseudopapilledema. Diagnostic performance was reported as the area under the curve (AUC), *p* value, optimal cut-off point, sensitivity, specificity, positive predictive value (PPV), negative predictive value (NPV), and 95% confidence intervals (CIs). For ROC curve analyses, one eye was randomly selected from each participant to ensure that each participant contributed only one eye and to avoid inter-eye correlation. Statistical significance was defined as a two-sided *p* < 0.05.

## Results

The median ages of patients with papilledema, pseudopapilledema, and healthy controls (HCs) did not differ significantly among the groups (*p* = 0.588). There was also no significant difference in sex distribution (*p* = 0.268).

In the papilledema group, 39 eyes (75%) had Frisén grade 1 papilledema, 9 eyes (17.3%) had grade 2, and 4 eyes (7.7%) had grade 3. Among patients with papilledema, 46.2% were receiving acetazolamide, 23.1% were receiving topiramate, 15.4% were receiving acetazolamide plus topiramate, and 11.5% were receiving acetazolamide combined with anticoagulant therapy.

Best-corrected visual acuity (BCVA) did not differ significantly between the papilledema and pseudopapilledema groups; however, BCVA was significantly lower in all patient groups compared with healthy controls (*p* < 0.001).

The mean optic disc area was significantly smaller in the pseudopapilledema without ODD group than in the ODD-related pseudopapilledema group (1.78 ± 0.37 mm² vs. 2.11 ± 0.50 mm², *p* < 0.001). Visible optic disc drusen were identified ophthalmoscopically in six eyes. Among the 54 eyes with ODD for which EDI-OCT images were available, PHOMS were detected in 16 eyes (29.6%), whereas a hyporeflective core surrounded by a hyperreflective border was identified in three eyes (5.6%). Demographic and clinical characteristics are summarized in Table 1.

**Table 1:** Demographic and clinical characteristics of the study participants

	ODD-related pseudopapilledema (*n* = 30 patients)	Pseudopapilledema without ODD (*n* = 32 patients)	Papilledema (*n* = 26 patients)	Healthy controls (*n* = 27 patients)	Total	Test statistics	*P* value
Age(years)	13 (4–17)	13 (7–17)	14 (6–17)	13 (5–17)	14 (4–17)	1.924	0.588^x^
Sex							
Female (n)	15 (50%)	22 (68.8%)	18 (69.2%)	14 (51.9%)	69 (60%)	3.888	0.268^y^
Male (n)	15 (50%)	10 (31.3%)	8 (30.8%)	13 (48.1%)	46 (40%)		
BCVA (LogMAR)	0.06 ± 0.11^b^	0.03 ± 0.06^b^	0.07 ± 0.1^b^	0 ± 0^a^			**< 0.001** ^**z**^

^x^Kruskal Wallis H Test; ^y^ Fisher’s Exact Test with Monte Carlo correction; ^z^ Generalized Estimating Equations; Median (Minimum – Maximum); n (%); Mean±Standard deviation

^a−c^: Groups sharing the same superscript letter are not significantly different

ODD, optic disc drusen; BCVA, best corrected visual acuity; LogMAR, Logarithm of the Minimum Angle of Resolution

### Peripapillary retinal nerve fiber layer thickness

The mean peripapillary RNFL (pRNFL) thickness was 129.85 ± 51.08 μm in the papilledema group, 128.22 ± 36.78 μm in the ODD-related pseudopapilledema group, 106.64 ± 17.10 μm in the pseudopapilledema without ODD group, and 99.89 ± 11.29 μm in the healthy control group.

No significant differences in average or sectoral pRNFL thickness were observed between the papilledema and ODD-related pseudopapilledema groups (all *p* > 0.05). Compared with both healthy controls and the pseudopapilledema without ODD group, eyes with papilledema demonstrated significantly greater average pRNFL thickness as well as thicker superior, inferior, and temporal quadrants, whereas nasal pRNFL thickness did not differ significantly. Eyes with ODD also showed significantly greater average and sectoral pRNFL thickness than healthy controls. Compared with healthy controls, the pseudopapilledema without ODD group demonstrated significantly greater average and superior pRNFL thickness (Table 2).

**Table 2:** Peripapillary RNFL and macular GC-IPL thicknesses (µm) in study groups

	ODD-related pseudopapilledema (*n* = 60 eyes)	Pseudopapilledema without ODD (*n* = 64 eyes)	Papilledema (*n* = 52 eyes)	Healthy controls (*n* = 54 eyes)	*p* ^x^
Disc area (mm^2^)	2.11 ± 0.5^a^	1.78 ± 0.37^b^	2.02 ± 0.35^a^	1.79 ± 0.31^b^	< 0.001
RNFL (µm)					
average	128.22 ± 36.78^b^	106.64 ± 17.1^c^	129.85 ± 51.08^b^	99.89 ± 11.29^a^	**< 0.001**
superior	157.2 ± 51.86^b^	134.08 ± 19.01^c^	164.17 ± 69.2^b^	123.46 ± 13.71^a^	**< 0.001**
inferior	181.58 ± 73.38^a^	137.2 ± 36.05^b^	176.35 ± 77.14^a^	127.41 ± 20.78^b^	**< 0.001**
nasal	97.13 ± 42.17^b^	81.72 ± 17.28^a^	86.31 ± 51.05^ab^	76.35 ± 13.73^a^	**0.003**
temporal	78.8 ± 20.87^bc^	73.41 ± 22.26^ac^	92.9 ± 34.52^b^	70.04 ± 10.74^a^	**< 0.001**
GC-IPL(µm)					
average	84.29 ± 11.46	84.74 ± 5.43	83.86 ± 6.04	84.06 ± 5.16	0.923
superior hemisphere	84.91 ± 10.89	85.33 ± 5.66	84.19 ± 5.88	84.53 ± 5.03	0.836
inferior hemisphere	82.98 ± 13.02	84 ± 5.84	83.48 ± 7.03	84.82 ± 5.44	0.642
superotemporal	83.37 ± 12.01	83.94 ± 5.3	82.67 ± 6.65	82.96 ± 5.12	0.806
inferotemporal	81.98 ± 14.32	84.11 ± 5.69	83.72 ± 7.55	84.33 ± 7.2	0.766
superior	85.85 ± 10.6	85.29 ± 6.3	84.56 ± 6.7	84.63 ± 5.52	0.834
inferior	81.78 ± 13.11	82.54 ± 6.77	82.25 ± 8.28	84.19 ± 4.84	0.355
superionasal	86.1 ± 11.62	86.83 ± 6.61	85.03 ± 7.01	85.09 ± 5.67	0.553
inferonasal	84.54 ± 12.05	85.43 ± 6.43	84.5 ± 7.52	84.19 ± 4.53	0.808

^x^Generalized Estimating Equations (GEE); Mean ± Standard deviation; ^a−c^:Groups sharing the same superscript letter are not significantly different

Values are presented at the eye level; both eyes were included when eligible

pRNFL, peripapillary retinal nerve fiber layer; GCIPL, ganglion cell inner plexiform layer; superiorhemi, superior hemisphere; inferiorhemi, inferior hemisphere

### Macular ganglion cell–inner plexiform layer thickness

No significant differences were found in average or sectoral macular GC-IPL thickness among the papilledema, ODD-related pseudopapilledema, pseudopapilledema without ODD, and healthy control groups (all p values > 0.05) (Table 2).

Within the papilledema group, neither mean pRNFL thickness nor mean GC-IPL thickness was significantly correlated with visual field mean deviation (*r* = 0.209, *p* = 0.251, and *r* = − 0.145, *p* = 0.428, respectively).

### Diagnostic performance of OCT parameters

Receiver operating characteristic (ROC) curve analyses were performed using one randomly selected eye from each participant to avoid inter-eye correlation. The diagnostic performance of conventional pRNFL parameters varied according to the comparison groups (Table 3). For papilledema versus healthy controls, inferior RNFL thickness demonstrated the highest discriminatory ability (AUC = 0.848, *p* < 0.001). For ODD-related pseudopapilledema versus healthy controls, inferior RNFL thickness showed the highest discriminatory ability (AUC = 0.906, *p* < 0.001). In pseudopapilledema without ODD versus healthy controls, superior RNFL thickness demonstrated significant discrimination (AUC = 0.720, *p* = 0.001). For papilledema versus ODD-related pseudopapilledema, nasal RNFL thickness showed modest discriminatory ability (AUC = 0.699, *p* = 0.005). For papilledema versus pseudopapilledema without ODD, inferior RNFL thickness demonstrated the highest discriminatory ability (AUC = 0.732, *p* < 0.001), followed by temporal RNFL thickness (AUC = 0.710, *p* = 0.002) (Table 3).

**Table 3:** Receiver operating characteristic (ROC) analysis of pRNFL measurements for differentiating papilledema and pseudopapilledema

	AUC (95% CI)	*P* value	Cut-off point (µm)	Sensitivity	Specificity	PPV	NPV
**Papilledema vs. HCs**							
Average RNFL	0.760 (0.626–0.894)	< 0.001	≥ 105	76.92%	77.78%	76.92%	77.78%
Superior RNFL	0.752 (0.609–0.895)	0.001	≥ 137	61.54%	92.59%	88.89%	71.43%
Inferior RNFL	0.848 (0.741–0.956)	< 0.001	≥ 128	84.62%	74.07%	75.86%	83.33%
Nasal RNFL	0.509 (0.349–0.668)	0.917	–	–	–	–	–
Temporal RNFL	0.788 (0.665–0.912)	< 0.001	≥ 74	73.08%	74.07%	73.08%	74.07%
**ODD-related pseudopapilledema vs. HCs**							
Average RNFL	0.833 (0.723–0.943)	< 0.001	≥ 104	86.67%	74.07%	78.79%	83.33%
Superior RNFL	0.790 (0.660–0.920)	< 0.001	≥ 142	63.33%	100%	100%	71.05%
Inferior RNFL	0.906 (0.825–0.988)	< 0.001	≥ 136	86.67%	88.89%	89.66%	85.71%
Nasal RNFL	0.706 (0.569–0.843)	0.003	≥ 91	53.33%	92.59%	88.89%	64.10%
Temporal RNFL	0.680 (0.540–0.820)	0.012	≥ 83	40.00%	92.59%	85.71%	58.14%
**Pseudopapilledema without ODD vs. HCs**							
Average RNFL	0.580 (0.431–0.730)	0.292	–	–	–	–	–
Superior RNFL	0.720 (0.587–0.853)	0.001	≥ 128	62.50%	81.48%	80.00%	64.71%
Inferior RNFL	0.606 (0.460–0.753)	0.153	–	–	–	–	–
Nasal RNFL	0.580 (0.432–0.728)	0.291	–	–	–	–	–
Temporal RNFL	0.535 (0.384–0.686)	0.652	–	–	–	–	–
**Papilledema vs. ODD-related pseudopapilledema**							
Average RNFL	0.578 (0.423–0.732)	0.324	–	–	–	–	–
Superior RNFL	0.515 (0.358–0.672)	0.854	–	–	–	–	–
Inferior RNFL	0.597 (0.446–0.749)	0.207	–	–	–	–	–
Nasal RNFL	0.699 (0.561–0.838)	0.005	≤ 90	88.46%	53.33%	62.16%	84.21%
Temporal RNFL	0.617 (0.467–0.766)	0.125	–	–	–	–	–
**Papilledema vs. Pseudopapilledema without ODD**							
Average RNFL	0.687 (0.547–0.826)	0.009	≥ 105	76.92%	56.25%	58.82%	75.00%
Superior RNFL	0.620 (0.464–0.776)	0.133	–	–	–	–	–
Inferior RNFL	0.732 (0.602–0.862)	< 0.001	≥ 126	88.46%	50.00%	58.97%	84.21%
Nasal RNFL	0.586 (0.435–0.737)	0.265	–	–	–	–	–
Temporal RNFL	0.710 (0.575–0.845)	0.002	≥ 68	88.46%	56.25%	62.16%	85.71%

ROC analyses were performed using one randomly selected eye per participant. PPV, positive predictive value; NPV, negative predictive value; ODD, optic disc drusen; HCs, healthy controls; CI, confidence interval

For non-significant ROC analyses, no optimal cut-off was identified; therefore, sensitivity, specificity, PPV, and NPV were not reported

ROC analyses of macular GC-IPL parameters showed no significant discriminatory ability between papilledema and healthy controls or between pseudopapilledema without ODD and healthy controls. In contrast, several GC-IPL parameters significantly discriminated ODD-related pseudopapilledema from healthy controls, with inferonasal GC-IPL showing the highest discriminatory ability (AUC = 0.731, *p* = 0.004). Multiple GC-IPL parameters also demonstrated significant discrimination between papilledema and ODD-related pseudopapilledema, with superotemporal GC-IPL showing the highest AUC (0.769, *p* = 0.001). No significant discrimination was observed between papilledema and pseudopapilledema without ODD (Table 4).

**Table 4:** Receiver operating characteristic (ROC) analysis of macular GC-IPL measurements for differentiating papilledema and pseudopapilledema

	AUC (95% CI)	*P* value	Cut-off point (µm)	Sensitivity	Specificity	PPV	NPV
**Papilledema vs. HCs**							
Average GC-IPL	0.508 (0.342–0.675)	0.921	–	–	–	–	–
Superior hemifield GC-IPL	0.553 (0.385–0.721)	0.536	–	–	–	–	–
Inferior hemifield GC-IPL	0.572 (0.406–0.737)	0.396	–	–	–	–	–
Superotemporal GC-IPL	0.608 (0.440–0.777)	0.209	–	–	–	–	–
Inferotemporal GC-IPL	0.545 (0.376–0.714)	0.600	–	–	–	–	–
Superior GC-IPL	0.592 (0.414–0.769)	0.311	–	–	–	–	–
Inferior GC-IPL	0.636 (0.465–0.806)	0.119	–	–	–	–	–
Superonasal GC-IPL	0.530 (0.348–0.711)	0.747	–	–	–	–	–
Inferonasal GC-IPL	0.513 (0.331–0.696)	0.886	–	–	–	–	–
**ODD-related pseudopapilledema vs. HCs**							
Average GC-IPL	0.699 (0.541–0.857)	0.013	≥ 88	70.00%	70.37%	63.64%	76.00%
Superior hemifield GC-IPL	0.642 (0.476–0.807)	0.093	–	–	–	–	–
Inferior hemifield GC-IPL	0.581 (0.408–0.753)	0.359	–	–	–	–	–
Superotemporal GC-IPL	0.682 (0.523–0.842)	0.025	≥ 84	75.00%	59.26%	57.69%	76.19%
Inferotemporal GC-IPL	0.595 (0.427–0.763)	0.266	–	–	–	–	–
Superior GC-IPL	0.651 (0.484–0.818)	0.076	–	–	–	–	–
Inferior GC-IPL	0.644 (0.473–0.816)	0.099	–	–	–	–	–
Superonasal GC-IPL	0.681 (0.524–0.839)	0.023	≥ 83	80.00%	48.15%	53.33%	76.47%
Inferonasal GC-IPL	0.731 (0.574–0.889)	0.004	≥ 88	65.00%	81.48%	72.22%	75.86%
**Pseudopapilledema without ODD vs. HCs**							
Average GC-IPL	0.569 (0.394–0.743)	0.441	–	–	–	–	–
Superior hemifield GC-IPL	0.547 (0.371–0.722)	0.601	–	–	–	–	–
Inferior hemifield GC-IPL	0.578 (0.401–0.756)	0.386	–	–	–	–	–
Superotemporal GC-IPL	0.553 (0.376–0.730)	0.555	–	–	–	–	–
Inferotemporal GC-IPL	0.521 (0.346–0.695)	0.816	–	–	–	–	–
Superior GC-IPL	0.549 (0.363–0.735)	0.605	–	–	–	–	–
Inferior GC-IPL	0.578 (0.398–0.759)	0.395	–	–	–	–	–
Superonasal GC-IPL	0.654 (0.477–0.830)	0.087	–	–	–	–	–
Inferonasal GC-IPL	0.637 (0.452–0.822)	0.146	–	–	–	–	–
**Papilledema vs. ODD-related pseudopapilledema**							
Average GC-IPL	0.706 (0.538–0.873)	0.016	≤ 87	77.27%	70.00%	73.91%	73.68%
Superior hemifield GC-IPL	0.708 (0.543–0.873)	0.014	≤ 86	77.27%	65.00%	70.83%	72.22%
Inferior hemifield GC-IPL	0.684 (0.510–0.858)	0.038	≤ 85	68.18%	75.00%	75.00%	68.18%
Superotemporal GC-IPL	0.769 (0.616–0.923)	0.001	≤ 86	94.44%	50.00%	63.04%	90.91%
Inferotemporal GC-IPL	0.725 (0.557–0.893)	0.009	≤ 88	94.44%	50.00%	63.04%	90.91%
Superior GC-IPL	0.738 (0.576–0.899)	0.004	≤ 88	94.44%	45.00%	60.71%	90.00%
Inferior GC-IPL	0.718 (0.541–0.896)	0.016	≤ 85	88.89%	65.00%	69.57%	86.67%
Superonasal GC-IPL	0.694 (0.524–0.865)	0.026	≤ 85	66.67%	70.00%	66.67%	70.00%
Inferonasal GC-IPL	0.715 (0.548–0.882)	0.012	≤ 86	72.22%	65.00%	65.00%	72.22%
**Papilledema vs. Pseudopapilledema without ODD**							
Average GC-IPL	0.543 (0.356–0.729)	0.653	–	–	–	–	–
Superior hemifield GC-IPL	0.608 (0.423–0.794)	0.252	–	–	–	–	–
Inferior hemifield GC-IPL	0.509 (0.317–0.701)	0.924	–	–	–	–	–
Superotemporal GC-IPL	0.678 (0.493–0.863)	0.060	–	–	–	–	–
Inferotemporal GC-IPL	0.590 (0.395–0.785)	0.367	–	–	–	–	–
Superior GC-IPL	0.675 (0.490–0.859)	0.063	–	–	–	–	–
Inferior GC-IPL	0.507 (0.303–0.710)	0.950	–	–	–	–	–
Superonasal GC-IPL	0.665 (0.477–0.853)	0.085	–	–	–	–	–
Inferonasal GC-IPL	0.637 (0.445–0.830)	0.163	–	–	–	–	–

ROC analyses were performed using one randomly selected eye per participant. PPV, positive predictive value; NPV, negative predictive value; HCs, healthy controls; ODD, optic disc drusen; CI, confidence interval

For non-significant ROC analyses, no optimal cut-off was identified; therefore, sensitivity, specificity, PPV, and NPV were not reported

## Discussion

In this study, we evaluated the diagnostic performance of conventional OCT-derived peripapillary RNFL and macular GC-IPL measurements for differentiating papilledema from pseudopapilledema in a pediatric population. The principal findings were that conventional RNFL measurements demonstrated limited diagnostic performance for distinguishing papilledema from ODD-related pseudopapilledema, whereas inferior RNFL thickness showed the highest discriminatory ability for differentiating papilledema from pseudopapilledema without ODD. In addition, macular GC-IPL measurements did not significantly differ among the study groups and showed limited diagnostic utility. These findings suggest that conventional OCT parameters alone may be insufficient for reliably distinguishing pediatric papilledema from pseudopapilledema and should be interpreted in conjunction with clinical evaluation and complementary imaging modalities.

Our findings demonstrated significantly increased average and sectoral pRNFL thicknesses in both papilledema and ODD-related pseudopapilledema compared with healthy controls. However, no significant differences were observed between the papilledema and ODD-related pseudopapilledema groups. This finding is consistent with previous studies demonstrating that both papilledema and ODD are associated with increased RNFL thickness, resulting in substantial overlap between these entities and limiting the diagnostic specificity of conventional OCT measurements [8–11, 17]. The American Academy of Ophthalmology report by Chang et al. also emphasized that although peripapillary RNFL measurements are valuable for identifying optic nerve head swelling, they should not be used in isolation to distinguish papilledema from pseudopapilledema because of the considerable overlap in RNFL thickness among these conditions [8]. In contrast, eyes with pseudopapilledema without ODD demonstrated only mild RNFL thickening, mainly involving the superior quadrant. Overall, our findings are consistent with previous reports suggesting that conventional pRNFL measurements are more useful for identifying optic disc swelling than for differentiating papilledema from ODD-related pseudopapilledema [8, 18]. 

ROC analysis further demonstrated that the diagnostic performance of conventional pRNFL measurements depended on the clinical comparison being made. Although nasal pRNFL thickness yielded the highest discriminatory ability for differentiating papilledema from ODD-related pseudopapilledema, the overall diagnostic performance remained modest (AUC = 0.699). This finding reflects the substantial structural overlap between these two conditions, both of which are characterized by optic nerve head elevation and increased peripapillary RNFL thickness. Similar limitations have been reported in previous studies, which showed that conventional OCT parameters alone cannot reliably distinguish papilledema from ODD and should be interpreted together with clinical findings and complementary imaging modalities [6, 8, 9, 17]. In contrast, inferior pRNFL thickness demonstrated the highest discriminatory performance for differentiating papilledema from pseudopapilledema without ODD (AUC = 0.732), followed by temporal pRNFL thickness (AUC = 0.710), suggesting that conventional OCT measurements may provide greater diagnostic value when optic disc drusen are absent. Overall, the ROC findings further support the concept that conventional OCT-derived RNFL measurements should be regarded as complementary rather than definitive tools in the evaluation of pediatric optic disc swelling.

In the present study, neither average nor sectoral macular GC-IPL thickness differed significantly among the papilledema, ODD-related pseudopapilledema, pseudopapilledema without ODD, and healthy control groups. However, ROC analysis demonstrated significant discriminatory ability for several regional GC-IPL parameters in specific pairwise comparisons. Inferonasal GC-IPL showed the highest discriminatory ability for ODD-related pseudopapilledema versus healthy controls (AUC = 0.731), whereas superotemporal GC-IPL showed the highest discriminatory ability for papilledema versus ODD-related pseudopapilledema (AUC = 0.769). These findings suggest that although GC-IPL thickness does not show consistent group-level differences, selected regional measurements may provide additional discriminatory information in specific clinical comparisons. Nevertheless, the moderate magnitude of these AUC values indicates that GC-IPL measurements alone are unlikely to provide sufficient diagnostic discrimination. These findings are in agreement with previous studies reporting that GC-IPL thickness is generally preserved in patients with mild papilledema and pseudopapilledema, limiting its usefulness for differential diagnosis [13, 16]. Preservation of GC-IPL thickness is biologically plausible, as ganglion cell loss is not expected during the early stages of optic disc swelling, when structural changes are predominantly confined to the peripapillary retinal nerve fiber layer [14, 15]. Therefore, although GC-IPL measurements may be valuable for detecting chronic neuronal loss during long-term follow-up, they appear to have limited utility in the initial differentiation of pediatric papilledema and pseudopapilledema.

The present study has several strengths. To our knowledge, few previous pediatric studies have evaluated the diagnostic performance of both peripapillary RNFL and macular GC-IPL measurements across four clinically relevant groups, including papilledema, ODD-related pseudopapilledema, pseudopapilledema without ODD, and healthy controls. In addition, the use of generalized estimating equation models allowed adjustment for the correlation between fellow eyes, thereby providing more robust statistical comparisons. Finally, the inclusion of ROC curve analyses enabled a comprehensive assessment of the diagnostic performance of conventional OCT parameters beyond simple group comparisons.

This study has several limitations. First, its retrospective single-center design may limit the generalizability of the findings. Second, enhanced depth imaging OCT was not available for all participants; therefore, ODD classification relied primarily on B-scan ultrasonography, with fundus autofluorescence and available EDI-OCT findings serving as complementary imaging modalities. Although this approach reflects routine clinical practice, the absence of standardized EDI-OCT imaging in all patients may have influenced diagnostic classification. Third, Bruch’s membrane opening configuration, which has been reported as a useful parameter for differentiating papilledema from pseudopapilledema, was not evaluated. Finally, patients with severe papilledema (Frisén grades 4–5) were not included. However, the primary objective of this study was to evaluate the diagnostic performance of conventional OCT parameters in clinically challenging cases in which papilledema and pseudopapilledema may overlap. Advanced papilledema is usually distinguishable on clinical fundus examination because of characteristic features such as marked optic disc swelling, peripapillary hemorrhages, vascular obscuration, and cotton-wool spots. Therefore, the exclusion of severe papilledema allowed the study to focus on the subgroup in which OCT has the greatest potential clinical value.

Conventional OCT-derived RNFL measurements provide useful quantitative information for the evaluation of pediatric optic disc swelling but have limited ability to reliably distinguish papilledema from pseudopapilledema, particularly in eyes with optic disc drusen. Although macular GC-IPL thickness did not differ significantly at the group level, selected regional GC-IPL parameters demonstrated discriminatory ability in specific comparisons. Therefore, conventional OCT findings should be interpreted in conjunction with clinical assessment, longitudinal follow-up, and complementary imaging modalities when evaluating children with suspected papilledema.

## Data Availability

No datasets were generated or analysed during the current study.
